# Painful Diabetic Neuropathy Is Associated with Compromised Microglial IGF-1 Signaling Which Can Be Rescued by Green Tea Polyphenol EGCG in Mice

**DOI:** 10.1155/2022/6773662

**Published:** 2022-02-22

**Authors:** Xin Chen, Yue Le, Si-Qi Tang, Wan-you He, Jian He, Yun-hua Wang, Han-bing Wang

**Affiliations:** ^1^Department of Anesthesiology, The First People's Hospital of Foshan, 81# North of Ling Nan Road, Foshan, 528000 Guangdong, China; ^2^Department of Anesthesiology, Nan Fang Hospital, Southern Medical University, Guangzhou, 510515 Guangdong, China; ^3^Department of Anesthesiology, Renmin Hospital of Wuhan University, Wuhan, 430060 Hubei, China

## Abstract

**Background:**

Painful diabetic neuropathy (PDN) is a frequent and troublesome complication of diabetes, with little effective treatment. PDN is characterized by specific spinal microglia-mediated neuroinflammation. Insulin-like growth factor 1 (IGF-1) primarily derives from microglia in the brain and serves a vital role in averting the microglial transition into the proinflammatory M1 phenotype. Given that epigallocatechin-3-gallate (EGCG) is a potent anti-inflammatory agent that can regulate IGF-1 signaling, we speculated that EGCG administration might reduce spinal microglia-related neuroinflammation and combat the development of PDN through IGF-1/IGF1R signaling.

**Methods:**

Type 1 diabetes mellitus (T1DM) was established by a single intraperitoneal (i.p.) injection of streptozotocin (STZ) in mice. The protein expression level of IGF-1, its receptor IGF1R, interleukin 1*β* (IL-1*β*), tumor necrosis factor-*α* (TNF-*α*), and inducible nitric oxide synthase (iNOS) was determined by Western blot or immunofluorescence.

**Results:**

The spinal IGF-1 expression markedly decreased along with the presence of pain-like behaviors, the spinal genesis of neuroinflammation (increased IL-1*β*, TNF-*α*, and Iba-1^+^ microglia), and the intensified M1 microglia polarization (increased iNOS^+^Iba-1^+^ microglia) in diabetic mice. IGF-1 could colocalize with neurons, astrocytes, and microglia, but only microglial IGF-1 was repressed in T1DM mice. Furthermore, we found that i.t. administration of mouse recombinant IGF-1 (rIGF-1) as well as i.t. or i.p. treatment with EGCG alleviated the diabetes-induced pain-like behaviors, reduced neuroinflammation (suppressed IL-1*β*, TNF-*α*, and Iba-1^+^ microglia), prevented the M1 microglia polarization (less iNOS^+^Iba-1^+^ microglia), and restored the microglial IGF-1 expression.

**Conclusions:**

Our data highlighted the importance of maintaining spinal IGF-1 signaling in treating microglia-related neuroinflammation in PDN. This study also provides novel insights into the neuroprotective mechanisms of EGCG against neuropathic pain and neuroinflammation through IGF-1 signaling, indicating that this agent may be a promising treatment for PDN in the clinical setting.

## 1. Introduction

Painful diabetic neuropathy (PDN) is a frequent and troublesome complication of diabetes, disturbing about one in four diabetic patients [1, 2]. The incidence of PDN is even rising over time [3], owing to the increasing diabetic population [4]. The characteristic clinical symptom of PDN comprises various unpleasant experiences such as mechanical allodynia [5], significantly reducing life quality and raising health care costs. Even opiates and antidepressants are employed for neuropathic pain; their utilization for PDN is limited due to the unbearable side effects and unsatisfactory response rates [6, 7]. Thus, investigation of applicable treatments for PDN is urgently needed.

Neuroinflammation in the spinal cord has been recognized as a vital process in neuropathic pain [8, 9]. One feature of neuroinflammation is the induction of activated microglia and reactive astrocytes that exaggerate the release of cytokines, chemokines, or toxic compounds [10, 11]. Specifically, researches on PDN found a comparable extent of neuroinflammation in the spinal cord and ascribe it to the activation of microglia rather than astrocytes [12, 13], indicating a specific role of microglia in PDN. The activated microglia include two functional phenotypes: the classically activated (M1) phenotype and the alternative activated (M2) phenotype [14, 15]. In response to pathogens or tissue injury, the resting (M0) microglia transits into the M1 phenotype that produces proinflammatory mediators (i.e., tumor necrosis factor-*α* (TNF-*α*) and interleukin 1 beta (IL-1*β*)) or toxic compounds [14, 15], whereas M2 microglia are associated with anti-inflammatory properties [14, 15]. Therefore, it was reasonable to assume that blocking the transition into M1 microglia or enhancing the M2 phenotype might attenuate PDN by reducing spinal neuroinflammation.

Insulin-like growth factor 1 (IGF-1) is an anabolic growth hormone providing a crucial role in brain growth, maturation, and neuroplasticity [16]. The IGF-1 receptor (IGF1R) expresses abundantly in the developing brain and remains highly active in the adult nervous system [17]. Clinical studies suggest that low IGF-1 levels are associated with diabetes and obesity [18–20]. It is also noted that IGF-1/IGF1R signaling has been implicated in microglia-mediated neuroinflammation. Some studies reported that IGF-1 mainly derives from microglia in comparison with astrocytes and neurons in the central nervous system [21, 22]. Further *in vitro* researches suggested direct anti-inflammatory effects of IGF-1 on microglia [23]. Several lines of evidence indicate that IGF-1 may also regulate the microglial phenotype: the increase of IGF-1 represses the M1 phenotype and promotes the M2 phenotype [22, 24]. A recent study also demonstrated that activating IGF1R with recombinant IGF-1 (rIGF-1) reduced neuroinflammation by enhancing M2 microglial polarization after cerebral hemorrhage in mice [20]. These results suggest that IGF-1/IGF1R signaling is a promising target for reducing spinal microglia-related neuroinflammation in PDN.

Green tea is a widely popular beverage, and its customary intake has gradually been reported with health benefits [25, 26]. (−)-Epigallocatechin-3-gallate (EGCG), the major bioactive component of green tea, exhibits robust antioxidant and neuroprotective effects against stroke [27] and neurodegenerative diseases [28]. EGCG supplementation can prevent the progression of diabetes [29] and alleviate maternal diabetes-induced neural dysfunction [30] in rodents. Interestingly, studies also demonstrated that EGCG is of strong anti-inflammatory properties by reducing microglia-induced neuroinflammation in the animal models of obesity [31] and stroke [27]. This anti-inflammatory effect of EGCG has been partly attributed to the action of suppressing neurotoxic M1 microglia and enhancing the anti-inflammatory M2 phenotype [20, 27]. EGCG is identified as a strong modulator of IGF-1/IGF1R signaling in cancer diseases [32]. However, the detailed effect of EGCG on IGF-1 and its associated microglia function in PDN remains to be defined. Hence, we speculated that EGCG administration might attenuate spinal microglia-related neuroinflammation and combat the development of PDN through IGF-1/IGF1R signaling.

In the present study, we established the diabetes model in C57BL/6J mice by streptozotocin (STZ) injection and showed that spinal IGF-1/IGF1R signaling and particularly microglial IGF-1 diminished along with pain-like behaviors, spinal neuroinflammation, and augmented M1 microglial polarization. These alterations could be relieved by intrathecal (i.t.) administration of recombinant IGF-1 (rIGF-1) and EGCG. Our data highlighted the importance of maintaining spinal IGF-1 signaling in treating microglia-related neuroinflammation in PDN. This study also provides novel insights into the neuroprotective mechanisms of EGCG against neuropathic pain and neuroinflammation through IGF-1 signaling, indicating that this agent may be a promising treatment for PDN in the clinical setting.

## 2. Materials and Methods

### 2.1. Animals

All experimental protocols and animal handling procedures were approved by Sun Yat-Sen University and conformed to the regulations on animal care accredited by the National Institutes of Health and the institutional animal ethical committee. Male C57BL/6J mice, weighing 25~30 g, were purchased from the Laboratory Animal Center of Guangdong Province (Guangzhou, China) and kept in a standard lab housing with a 12 h light/dark cycle at a temperature of 21 ± 2°C and 60–70% humidity and allowed access to standard diet and water *ad libitum*.

### 2.2. Induction of Diabetes in Mice

We induced diabetes by a single i.p. injection of freshly prepared streptozotocin (STZ) (150 mg/kg; Sigma-Aldrich, Germany) in sterile 0.1 M sodium citrate buffer (pH 4.5) [33]. Control mice were only injected i.p. with vehicle solution (sterile 0.1 M sodium citrate buffer, pH 4.5). The successful establishment of T1DM was confirmed by determining the fasting blood glucose level with Accu-Chek test strips (Roche Diagnostics, Indianapolis, IN, U.S.A.) 3 days after the STZ injection. All the STZ-injected mice presented with high levels of blood glucose (>16.7 mmol/L). No mouse died through the study period or required insulin supplementation to offset extreme weight loss, and at the study end, all STZ-injected mice remained hyperglycemic (fasting blood glucose > 16.7 mmol/L).

### 2.3. Drug Administration

The intrathecal injection method was performed as previously described [34, 35]. In brief, mice were covered with a soft towel and held gently but firmly by the hip bones via the thumb and index finger of the nondominant hand of the operator. A 5 *μ*L microsyringe (Hamilton Company, Nevada, USA; 30 G needle) was inserted at the midline of the iliac crest between the lumbar 5th and 6th vertebrae. This region is considered the level of the cauda equina. A reflexive flick of the tail indicates the successful puncture of the dura. Because this reaction and muscle tone are essential reflections, intrathecal injections were performed in conscious mice. After achieving a 90% to 95% success rate in training sessions, we started these experiments. According to our recent study [36], mouse rIGF-1 (Novoprotein, Shanghai, China) was diluted in saline and administered intrathecally (i.t.) 1 *μ*g/d from D3 (3 days after STZ injection) to D5 after STZ injection, while the sham group was i.t. injected with equal volume of saline. EGCG (Sigma-Aldrich, St. Louis, MO, USA) was diluted in saline and administered i.t. 2 *μ*g/d [27] or i.p. 20 mg/kg/d [37] from D3 to D5 after STZ injection.

### 2.4. Experimental Groups

Our study included three independent experimental stages: (1) Stage 1 is aimed at determining the pattern of spinal IGF-1/IGF1R signaling along with the development of PDN. (2) Stage 2 is intended to assess the effect of intrathecal rIGF-1 treatment on PDN and spinal microglia-related neuroinflammation. (3) Stage 3 examined the effect of EGCG treatment on PDN and the neuroinflammation associated with microglia and IGF-1 signaling. The below is the detailed protocol and timeline:

*Stage 1*: (1) control group: mice received the i.p. injection of vehicle solution (0.1 M sodium citrate buffer); (2) T1DM group: mice received the i.p. injection of STZ. The behavioral tests were performed on baseline (BL, 30 min before saline or STZ injection), D3, D7, D14, and D21 (*n* = 8/group). In addition, Western blot and immunofluorescence were performed on lumbar spinal cord tissues at D14 and D21 (*n* = 4 or 5/group).

*Stage 2*: (1) sham group: mice received the i.t. injection of saline from D3 to D5; (2) T1DM group: mice received the i.p. injection of STZ followed by i.t. injection of saline from D3 to D5; (3) rIGF-1 group: mice received the i.p. injection of STZ followed by i.t. injection of rIGF-1 (1 *μ*g/d) from D3 to D5. The behavioral tests were performed on BL, D7, D14, and D21 (*n* = 9/group). Western blot and immunofluorescence were conducted on lumbar spinal cord tissues at D14 (*n* = 5/group).

*Stage 3*: (1) sham group: mice received the i.t. injection of saline from D3 to D5; (2) T1DM group: mice received the i.p. injection of STZ followed by i.t. injection of saline from D3 to D5; (3) EGCG_i.t._ group: mice received the i.p. injection of STZ followed by i.t. injection of EGCG (2 *μ*g/d) from D3 to D5; (4) EGCG_i.p._ group: mice received the i.p. injection of STZ followed by i.p. injection of EGCG (20 mg/kg/d) from D3 to D5. The behavioral tests were performed on BL, D7, D14, and D21 (*n* = 9/group). Western blot and immunofluorescence were conducted on lumbar spinal cord tissues at D14 (*n* = 5/group).

### 2.5. Behavioral Tests

On the experiment day, the mice were placed individually in transparent test compartments with a wire mesh bottom and habituated for one hour. Paw mechanical withdrawal thresholds (PMWTs) in response to mechanical stimuli were evaluated using the electronic von Frey unit (Bioseb, Montpellier, France) with a flexible metal filament applying increasing force (from 0 to 10 g) against the plantar surface of the hind paw of the mouse [38]. The nocifensive paw withdrawal response automatically turned off the stimulus, and the mechanical pressure that evoked the response was recorded. Measurements were repeated 5 times, and the final value was obtained by averaging the 5 measurements.

Thermal hypersensitivity was assessed by measuring paw thermal withdrawal latency (PTWL) to thermal stimuli using the PL-200 Plantar Analgesia Tester (Chengdu Technology & Market Co., Ltd., Sichuan, China) as described previously [39, 40]. The mice were placed on a glass plate and were allowed to habituate to the apparatus for 30 min. The radiant heat lamp source was adjusted to position right beneath the hind paw's plantar surface, vertically projecting a light spot with a diameter of 5 mm. The PTWL was calculated by averaging three individual trials with 5 min intervals to avert unexpected thermal sensitization. A cutoff time of 12 s was set to avoid tissue injury.

### 2.6. Western Blot

The mice were sacrificed by anesthetic overdose (2.5% Avertin, 1600 mg/kg, i.p.). The lumbar enlargement (L4–L5) of the spinal cord was rapidly removed and homogenized in ice-cold RIPA buffer (Beyotime, Shanghai, China). The lysates were kept for 30 min and then centrifuged at 14,000 g for 10 min at 4°C. Supernatants were collected, and the protein concentration was determined by the BCA protein assay kit (Boster, Wuhan, China). Equal amounts of protein samples (50 *μ*g) from each group were separated using 10% sodium dodecyl sulfate-polyacrylamide gel electrophoresis and transferred onto polyvinylidene difluoride (Millipore, Bedford, MA, USA) membranes. Then, the membranes were blocked with 3% nonfat milk in TBST for 1 h at room temperature and then incubated with the primary antibodies overnight at 4°C. On the next day, the membranes were rinsed three times with TBST and incubated with secondary antibody (anti-mouse or rabbit IgG, 1 : 5000; Boster) for 2 h at room temperature. After washing three times with TBST, the protein bands were detected with enhanced chemiluminescent reagents (Beyotime) and analyzed in the Fluochem HD2 Imaging System (Alpha Innotech, USA). The densitometry of the blots was analyzed with ImageJ software (version 1.43) and normalized to *β*-actin. The experimenters were blinded to the group assignment.

The following primary antibodies were used: anti-Arg-1 (Cell Signaling Technology; 1 : 1000), anti-IGF-1 (Abclonal, Wuhan, China; 1 : 500), anti-IGF1R (Abclonal; 1 : 3000), anti-IL-1*β* (Abclonal; 1 : 3000), anti-iNOS (Abclonal; 1 : 1000), anti-p-IGF1R (Abclonal; 1 : 500), anti-TNF-*α* (Cell Signaling Technology; 1 : 1000), and anti-*β*-actin (Cell Signaling Technology; 1 : 1000). The experiments were carried out in triplicates (*n* = 5/group).

### 2.7. Immunofluorescence Analysis

For immunofluorescence analysis, mice were transcardially perfused with 4% iced formaldehyde (*n* = 4 or 5/group). After, the lumbar spinal cord was removed, embedded in paraffin, and cut into 5 *μ*m thick serial sections. After deparaffination, rehydration, and heat-induced antigen retrieval with microwave oven (microwave method), the sections were incubated with normal goat/donkey bovine serum for 60 min at room temperature and were then incubated overnight at 4°C with primary antibodies against calcitonin gene-related peptide (CGRP) (goat polyclonal, Abcam; 1 : 400), GFAP (mouse monoclonal, Cell Signaling Technology; 1 : 200), Iba-1 (mouse monoclonal, Millipore, Darmstadt, Germany; 1 : 200), IGF-1 (rabbit monoclonal, Abcam; 1 : 200), IGF1R (rabbit polyclonal, Abcam; 1 : 200), iNOS (rabbit polyclonal, Abclonal; 1 : 200), p-IGF1R (Tyr-1161) (rabbit polyclonal, Abclonal; 1 : 100), and NeuN (mouse monoclonal, Abcam; 1 : 200). The next day, the sections were washed by PBS and incubated for 60 min at room temperature with Dylight 488 (1 : 200; goat anti-rabbit or donkey anti-goat; Abcam) or Dylight 567-labeled goat anti-rabbit secondary antibody (1 : 400; Abcam). Nuclei were counterstained with 4′,6-diamidino-2-phenylindole (DAPI). The target area was the spinal dorsal horn (SDH). Images were acquired under immunofluorescence microscopy (Olympus, Tokyo, Japan), and the quantification of cell numbers was performed manually by counting the number of positive cells using Image Pro™ Plus software.

### 2.8. Statistical Analysis

All data are expressed as means ± standard deviations (SD). Statistical differences between various groups were analyzed using one-way analysis of variance (ANOVA), followed by the *post hoc* test using GraphPad Prism 5 (GraphPad Software Inc., USA).

## 3. Results

### 3.1. Spinal IGF-1/IGF1R Signaling Diminished along with the Development of Pain-Like Behaviors in STZ-Injected Diabetic Mice

**Figure 1:**
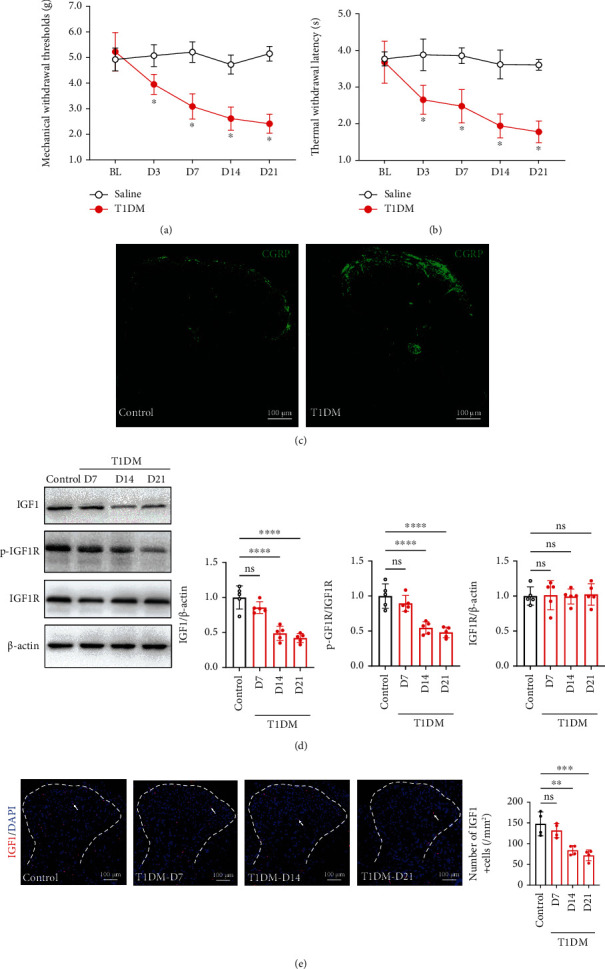
Spinal IGF-1/IGF1R signaling diminished along with the development of pain-like behaviors in STZ-injected diabetic mice. (a) The von Frey test showed that the T1DM (STZ-injected) mice displayed mechanical allodynia (reduction in PMWTs). The PMWTs were evaluated by the electronic von Frey test in vehicle (control) or STZ (T1DM)-treated mice. Data were presented as the mean ± SD. ^∗^*P* < 0.05 vs. control group. *n* = 8/group. (b) The T1DM mice developed thermal hypersensitivity (decrease in PTWL). The PTWL was assessed by a thermal paw stimulation system. Data were presented as the mean ± SD. ^∗^*P* < 0.05 vs. control group. *n* = 9/group. (c) Fluorescent graphs demonstrating CGRP expression in the SDH of control and T1DM mice on D14. Scale bar = 100 *μ*m. CGRP, a neuropeptide, is widely distributed in nociceptive circuits and is closely involved in pain transmission. (d) Western blot showing IGF1, p-IGF1R, and IGF1R in the lumbar spinal cord on D7, D14, and D21 after STZ injection. ^∗∗∗∗^*P* < 0.0001.*n* = 5/group. Data were presented as the mean ± SD. (e) Immunostaining of the SDH showing IGF1^+^ (red) cells in control and T1DM mice. The nuclei were counterstained with DAPI (blue). Scale bar = 100 *μ*m. ^∗∗^*P* < 0.01 or ^∗∗∗^*P* < 0.001. *n* = 4/group. Data were presented as the mean ± SD. BL: baseline; CGRP: calcitonin gene-related peptide; IGF-1: insulin-like growth factor 1; IGF1R: insulin-like growth factor 1 receptor; p-IGF1R: phospho-IGF1R (Tyr-1161); ns: not significant; control: the mice treated with vehicle (0.1 M sodium citrate buffer); SDH: the spinal dorsal horn; T1DM: the mice treated with STZ (diabetic mice).

We induced the diabetic model by injecting mice with a single dose of STZ (150 mg/kg, i.p.). To assess the time course of pain-like behaviors, we conducted the electronic von Frey test before and after the STZ injection. Our data showed that, compared with the vehicle (0.1 M sodium citrate buffer)-treated mice, the paw mechanical withdrawal thresholds (PMWTs) of T1DM (STZ-injected) mice began to decline on D3 (3 days after STZ injection) and reached the bottom on D14 and lasted to D21 (Figure 1(a); T1DM vs. control, *F* (1, 14) = 364.1, *P* < 0.0001, *n* = 8), signifying the development of mechanical allodynia in the T1DM mice. We also determined the thermal nociception behaviors after the STZ injection. We found that compared with the vehicle-treated mice, the paw thermal withdrawal latency (PTWL) of T1DM mice dropped after STZ injection (Figure 1(b); T1DM vs. control, *F* (1, 14) = 155.7, *P* < 0.0001, *n* = 8), indicating the occurrence of thermal hypersensitivity in T1DM mice. CGRP is a neuropeptide widely distributed in nociceptive circuits and is closely involved in pain transmission [41]. The immunostaining of the SDH suggested that the CGRP immunoreactivity of T1DM mice was more intense than that of control mice (Figure 1(c)).

Furthermore, we detected the IGF-1 and IGF1R expression in the lumbar spinal cord on D7, D14, and D21 after vehicle or STZ injection. The Western blot data showed that IGF-1 protein expression in the lumbar spinal cord of T1DM mice was substantially less than that of control mice on D14 and D21 (Figure 1(d), *P* < 0.0001, *n* = 5). Yet, the statistical significance in IGF1R was not observed (Figure 1(d), *P* > 0.05, *n* = 5). Given that IGF1R belongs to the large class of tyrosine kinase receptors and its activity is predominantly regulated by phosphorylation [42], we determined the level of IGF1R phosphorylation at Tyr-1161 (p-IGF1R). Consistently, we found that the level of p-IGF1R was reduced in the lumbar spinal cord of T1DM mice (Figure 1(d), *P* < 0.0001 on D14 and *P* < 0.0001 on D21, *n* = 5). Moreover, the number of IGF-1+ cells in the SDH of T1DM mice extensively decreased on D14 and D21 (Figure 1(e); T1DM-D14 and T1DM-D21 vs. control, 84.07 ± 11.23 and 71.71 ± 12.98 vs. 147.7 ± 28.59/mm^2^, *P* < 0.01 & *P* < 0.001, *n* = 4). All these data indicated that spinal IGF-1/IGF1R signaling downregulated parallel to the development of painful diabetic neuropathy (PDN).

### 3.2. Microglial IGF-1 in the SDH Declined in the SDH of STZ-Injected Diabetic Mice

**Figure 2:**
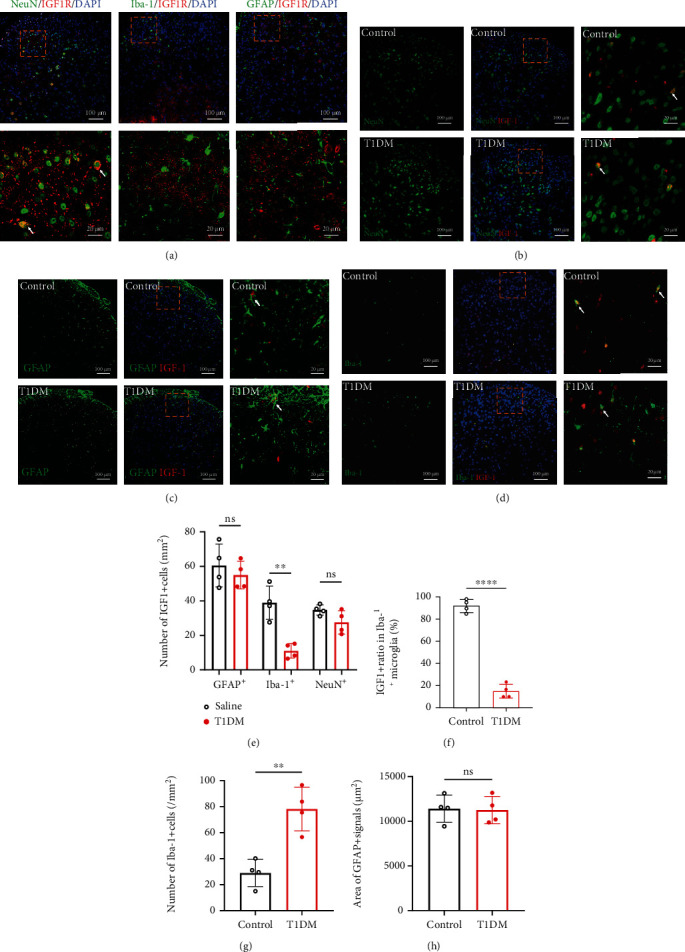
Microglial IGF-1 in the SDH declined in the SDH of STZ-injected diabetic mice. (a) Double immunofluorescence-labeling of IGF1R (red) with NeuN, GFAP, or Iba-1 (green) in the SDH of diabetic mice on D14. The scale bar of the upper panels is 100 *μ*m. The yellow boxes are zoomed-in in the below panels (scale bar = 20 *μ*m). The nuclei were counterstained with DAPI (blue). (b) Double-staining of IGF-1 (red) with NeuN (neuronal marker, green) in the SDH of diabetic mice on D14. The scale bar of the left and middle panels is 100 *μ*m. The yellow boxes are zoomed-in in the right panels (scale bar = 20 *μ*m). The nuclei were counterstained with DAPI (blue). (c) Double-staining of IGF-1 (red) with GFAP (astrocyte marker, green) in the SDH of diabetic mice on D14. The scale bar of the left and middle panels is 100 *μ*m. The yellow boxes are zoomed-in in the right panels (scale bar = 20 *μ*m). The nuclei were counterstained with DAPI (blue). (d) Double-staining of IGF-1 (red) with Iba-1 (microglial marker, green) in the SDH of diabetic mice on D14. The scale bar of the left and middle panels is 100 *μ*m. The yellow boxes are zoomed-in in the right panels (scale bar = 20 *μ*m). The nuclei were counterstained with DAPI (blue). (e) Quantitative analysis of GFAP immunoreactivity area in the SDH on D14. Data are expressed as the mean ± SD. *n* = 4/group. (e) The cell number of IGF1^+^NeuN^+^, IGF1^+^GFAP^+^, or IGF1^+^Iba-1^+^ in the SDH. ^∗∗^*P* < 0.01. Data are expressed as the mean ± SD. *n* = 4/group. (f) IGF-1^+^ ratio in Iba-1^+^ cells in the SDH on D14. ^∗∗∗∗^*P* < 0.0001. Data are expressed as the mean ± SD. *n* = 4/group. (g) The quantitative analysis of Iba-1^+^ microglia in the SDH on D14. ^∗∗^*P* < 0.01. Data are expressed as the mean ± SD. *n* = 4/group. (h) The area of GFAP^+^ immunoreactivity in the SDH on D14. Data are expressed as the mean ± SD. *n* = 4/group. GFAP: glial fibrillary acidic protein; Iba-1: ionized calcium-binding adaptor protein-1; IGF-1: insulin-like growth factor 1; IGF1R: insulin-like growth factor 1 receptor; ns: not significant; control: the mice treated with vehicle (0.1 M sodium citrate buffer); SDH: the spinal dorsal horn; T1DM: the mice treated with STZ (diabetic mice).

Then, we employed immunofluorescence double-labeling to examine the coexpression of IGF1R or IGF-1 with neurons (NeuN^+^), astrocytes (GFAP^+^), and microglia (Iba-1^+^) in the SDH on D14. As shown in Figure 2(a), IGF1R in the SDH of STZ-injected diabetic mice was primarily expressed on neurons and microglia (Figure 2(d)). IGF-1 in the SDH was expressed on neurons, astrocytes, and microglia. The comparison analysis suggested that compared with control mice, the number of IGF-1^+^ microglia was markedly less (Figures 2(b)–2(e); T1DM vs. control, 11.07 ± 4.264 vs. 38.92 ± 9.696/mm^2^, *P* = 0.0019; *n* = 4), while a significant difference in IGF-1^+^ neurons or astrocytes was not observed (Figures 2(b)–2(e); *P* = 0.4814 for GFAP^+^, *P* = 0.0999 for NeuN^+^; *n* = 4).

In addition, we analyzed the Iba-1^+^ microglia number and GFAP^+^ immunoreactivity area in the SDH of control and T1DM mice. We found that compared with control mice, the Iba-1^+^ microglia number in the SDH of T1DM mice was significantly higher (Figure 2(g); T1DM vs. control, 78.13 ± 16.77 vs. 28.99 ± 10.41/mm^2^, *P* = 0.0025; *n* = 4), whereas the difference in the GFAP^+^ immunoreactivity area was not significant (Figure 2(h), *P* = 0.6457; *n* = 4). Based on these results and the previous evidence that microglia are indispensable for spinal neuroinflammation in PDN [43], we postulated that the inhibition of IGF-1/IGF1R signaling might play a crucial role in spinal microglia activation of diabetic mice.

### 3.3. Activated M1 Phenotype Microglia and Neuroinflammation Surged in the SDH of STZ-Injected Diabetic Mice

**Figure 3:**
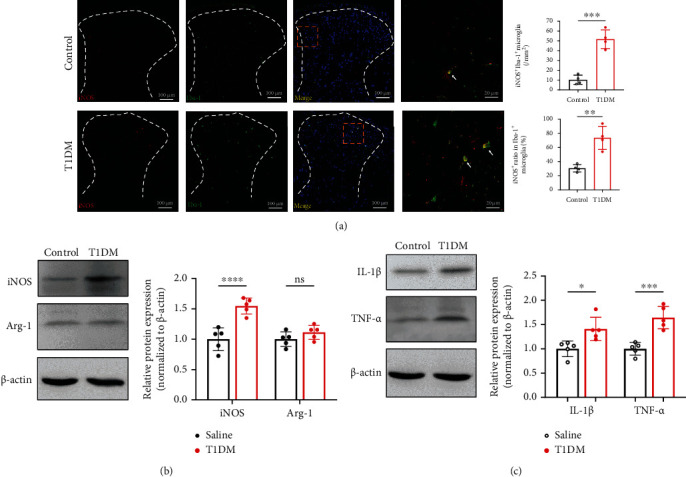
Activated M1 phenotype microglia and neuroinflammation surged in the SDH of STZ-injected diabetic mice. (a) Double-staining of iNOS (M1 phenotype microglia marker, red) with Iba-1 in the SDH on D14. The histograms represent the number and ratio of iNOS^+^Iba-1^+^ cells in the SDH, respectively. ^∗∗^*P* < 0.01 or^∗∗∗^*P* < 0.001. Data are expressed as the mean ± SD. *n* = 4/group. (b) Western blot indicating the protein expression of iNOS (M1 phenotype microglia marker) and Arg-1 (M2 phenotype microglia marker, red) in the spinal cord on D14. ^∗∗∗∗^*P* < 0.0001.*n* = 5/group. Data were presented as the mean ± SD. (c) Western blot showing the protein expression of IL-1*β* and TNF-*α* (proinflammatory cytokines) in the spinal cord on D14. ^∗∗^*P* < 0.01 or ^∗∗∗^*P* < 0.01. *n* = 5/group. Data were presented as the mean ± SD. Arg-1: arginase 1 (M2 phenotype microglia marker); IL-1*β*: interleukin 1*β*; iNOS: inducible nitric oxide synthase (M1 phenotype microglia marker); ns: not significant; TNF-*α*: tumor necrosis factor-*α*; control: the mice treated with vehicle (0.1 M sodium citrate buffer); SDH: the spinal dorsal horn; T1DM: the mice treated with STZ (diabetic mice).

The switch between M1 and M2 microglial phenotypes regulates inflammation in the central nervous system [44, 45]. Thus, we investigated the alteration of M1 (iNOS^+^) [46] and M2 (Arg-1^+^) [47] microglia phenotypes in the spinal cord. Our immunostaining results showed that the number and ratio of iNOS^+^Iba-1^+^ microglia increased in the SDH of T1DM mice compared with control mice (Figure 3(a); *P* = 0.002 for the number of iNOS^+^Iba-1^+^ cells, *P* = 0.0024 for iNOS^+^ ratio in Iba-1^+^ microglia, *n* = 4). In contrast, the Arg-1^+^Iba-1^+^ microglia were not detectable by immunostaining in the SDH of either control or T1DM mice. Thus, we sought to detect the protein expression of iNOS and Arg-1 by Western blot in the spinal cord. The data revealed that the iNOS protein expression was upregulated in T1DM mice on D14 (Figure 3(b); *P* = 0.0007, *n* = 5) while Arg-1 was not altered (Figure 3(b); *P* = 0.1708, *n* = 5), suggesting a significant increase of M1 microglia in the spinal cord of diabetic mice. M1 microglia are characterized by releasing proinflammatory cytokines such as IL-1*β* and TNF-*α*. The further detection of IL-1*β* and TNF-*α* corroborated the activation of M1 microglia by showing that IL-1*β* and TNF-*α* protein expressions are elevated in the T1DM mice (Figure 3(c); *P* = 0.0135 for IL-1*β*, *P* = 0.0006 for TNF-*α*, *n* = 5).

### 3.4. Intrathecal Treatment with rIGF-1 Relieved PDN and Prevented Spinal IGF-1/IGF1R Signaling Dysfunction

**Figure 4:**
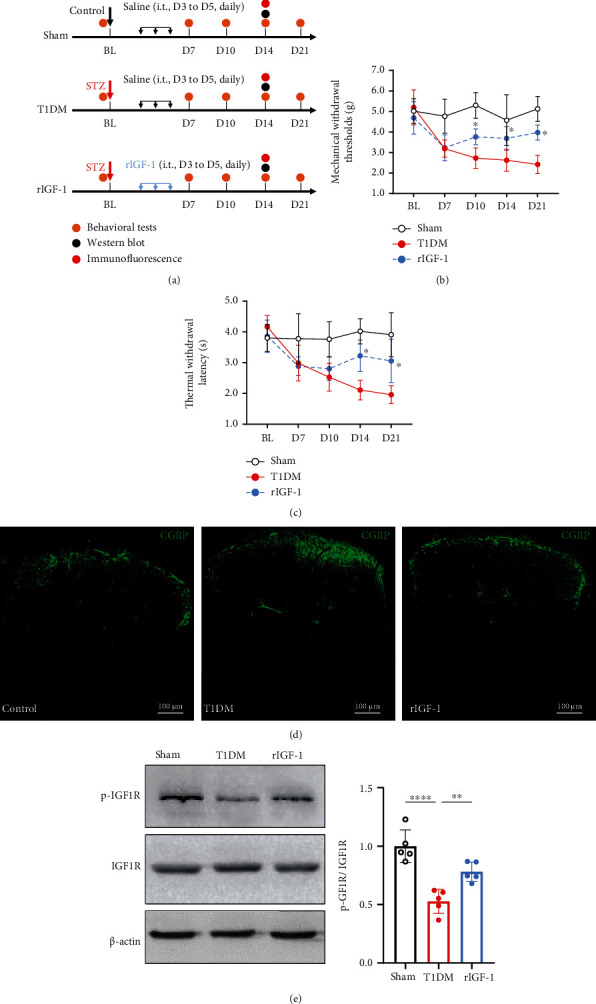
Intrathecal treatment with rIGF-1 relieved PDN and prevented spinal IGF-1/IGF1R signaling dysfunction. (a) Schematic illustration of the group assignment in the experimental stage. (b) Intrathecal treatment with recombinant IGF-1 (rIGF-1 group, 1 *μ*g/d, from D3 to D5 after STZ injection) reduced mechanical allodynia (rise in PMWTs) in T1DM mice. Data were presented as the mean ± SD. ^∗^*P* < 0.05 vs. the T1DM group. Two-way ANOVA followed by Bonferroni's post hoc test. *n* = 8/group. (c) Intrathecal treatment with recombinant IGF-1 attenuated thermal hypersensitivity (increase in PTWL) in the T1DM mice. Data were presented as the mean ± SD. ^∗^*P* < 0.05 vs. the T1DM group. Two-way ANOVA followed by Bonferroni's post hoc test. *n* = 8/group. (d) Fluorescent graphs demonstrating CGRP expression in the SDH on D14. Scale bar = 100 *μ*m. (e) Western blot showing the protein expression of p-IGF1R and IGF1R in the lumbar spinal cord on D14. ^∗∗^*P* < 0.01 or ^∗∗∗∗^*P* < 0.0001. *n* = 5/group. Data were presented as the mean ± SD. BL: baseline; CGRP: calcitonin gene-related peptide; IGF1R: insulin-like growth factor 1 receptor; p-IGF1R: phospho-IGF1R (Tyr-1161); rIGF-1: recombinant IGF-1; SDH: the spinal dorsal horn; T1DM: the mice treated with STZ (diabetic mice).

To confirm the role of spinal IGF-1/IGF1R signaling in PDN, we intrathecally (i.t.) injected T1DM mice with rIGF-1 (1 *μ*g/d, from D3 to D5 after STZ injection) (Figure 4(a)). The behavioral examinations indicated that i.t. treatment with rIGF-1 caused the recovery of PMWT and PTWL (Figures 4(b) and 4(c); for PMWT, *F* (1, 14) = 25.99, *P* = 0.0002, *n* = 8; for PTWL, *F* (1, 14) = 11.65, *P* = 0.0042, *n* = 8). Also, the increased CGRP immunoreactivity in the SDH of T1DM mice was reduced by the i.t. injection of rIGF-1 on D14 (Figure 4(d)). The further Western blot assay confirmed that the treatment with rIGF-1 enhanced the spinal p-IGF1R level in T1DM mice (Figure 4(e), *P* = 0.0067, *n* = 5).

### 3.5. Activating Spinal IGF-1 Signaling Maintained Microglial IGF-1 Expression and Reduced Neuroinflammation in the Spinal Cord of Diabetic Mice

**Figure 5:**
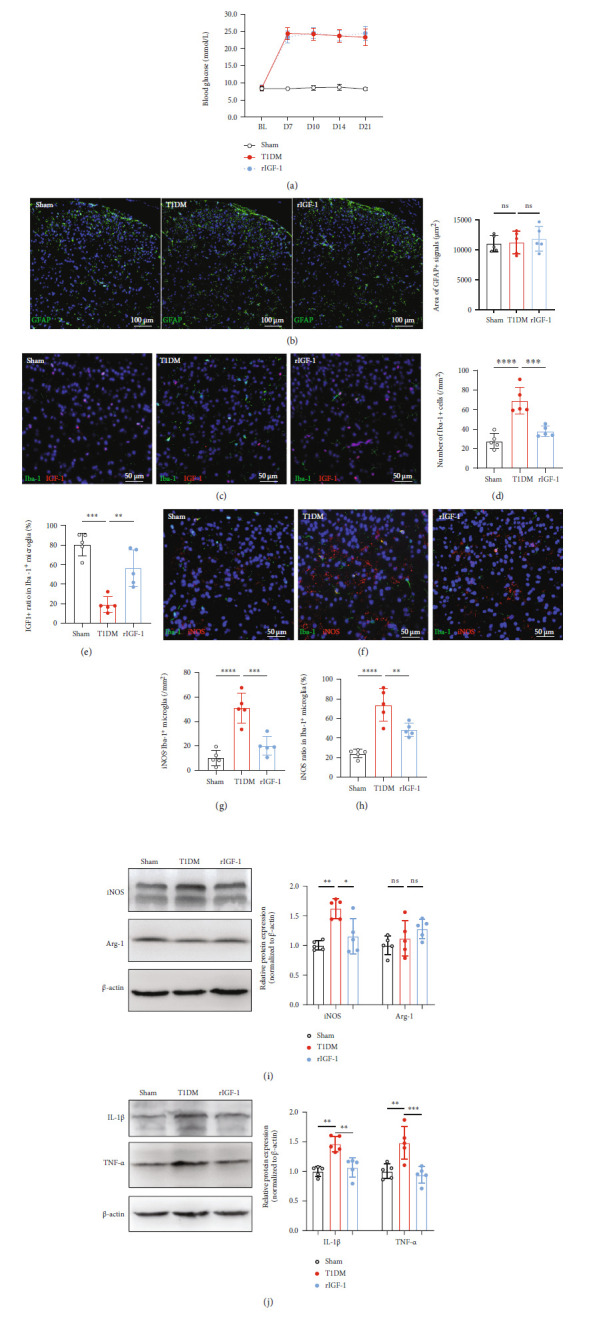
Activating spinal IGF-1 signaling maintained microglial IGF-1 expression and reduced neuroinflammation in the spinal cord of diabetic mice. (a) Time course of blood glucose concentration in the sham, T1DM, and rIGF-1 (i.t. treated with rIGF-1, 1 *μ*g/d, from D3 to D5 after STZ injection) groups. Data were presented as the mean ± SD. *n* = 8/group. (b) The area of GFAP immunoreactivity in the SDH of the sham, T1DM, and rIGF-1 groups on D14. *n* = 5/group. Data were presented as the mean ± SD. (c) Fluorescent graphs displaying the double-labeling of IGF-1 (red) with Iba-1 (green) in the SDH of the sham, T1DM, and rIGF-1 groups. Scale bar = 50 *μ*m. (d) The statistical analysis of Iba-1^+^ cells in the SDH of the sham, T1DM, and rIGF-1 groups. *n* = 5/group. Data were presented as the mean ± SD. ^∗∗∗^*P* < 0.001 or^∗∗∗∗^*P* < 0.0001. (e) The analysis of IGF-1^+^ ratio in Iba-1^+^ microglia in the SDH of the sham, T1DM, and rIGF-1 groups. *n* = 5/group. Data were presented as the mean ± SD. ^∗∗^*P* < 0.01 or ^∗∗∗∗^*P* < 0.0001. (f) Fluorescent graphs showing the double-labeling of iNOS (red) with Iba-1 (green) in the SDH of the sham, T1DM, and rIGF-1 groups. Scale bar = 50 *μ*m. (g) The statistical analysis of iNOS^+^Iba-1^+^ cells in the SDH of the sham, T1DM, and rIGF-1 groups. *n* = 5/group. Data were presented as the mean ± SD. ^∗∗∗^*P* < 0.001 or^∗∗∗∗^*P* < 0.0001. (h) The analysis of iNOS^+^ ratio in Iba-1^+^ microglia in the SDH of the sham, T1DM, and rIGF-1 groups. *n* = 5/group. Data were presented as the mean ± SD. ^∗∗^*P* < 0.01 or^∗∗∗∗^*P* < 0.0001. (i) Western blot showing the protein expression of iNOS and Arg-1 in the lumbar spinal cord on D14. ^∗∗^*P* < 0.01 or^∗∗∗∗^*P* < 0.0001. *n* = 5/group. Data were presented as the mean ± SD. (j) Western blot demonstrating the protein expression of IL-1*β* and TNF-*α* in the lumbar spinal cord on D14. ^∗∗^*P* < 0.01 or^∗∗∗^*P* < 0.001.*n* = 5/group. Data were presented as the mean ± SD. Arg-1: arginase 1 (M2 phenotype microglia marker); IL-1*β*: interleukin 1*β*; iNOS: inducible nitric oxide synthase (M1 phenotype microglia marker); ns: not significant; rIGF-1: recombinant IGF-1; TNF-*α*: tumor necrosis factor-*α*; SDH: the spinal dorsal horn; T1DM: the mice treated with STZ (diabetic mice).

We found that the i.t. treatment with rIGF-1 did not alter the blood glucose level of T1DM mice (Figure 5(a), *F*(1, 14) = 0.1382, *P* = 0.7157, *n* = 8). Treatment with rIGF-1 did not affect the GFAP immunoreactivity (Figure 5(b), *P* = 0.8236, *n* = 5) but reduced the number of Iba-1^+^ microglia in the SDH of T1DM mice on D14 (Figures 5(c) and 5(d), *P* = 0.0005, *n* = 5). Noticeably, treatment with rIGF-1 increased the IGF-1^+^ ratio in Iba-1^+^ cells (Figures 5(c) and 5(e), *P* = 0.0018, *n* = 5) and reduced iNOS^+^Iba-1^+^ microglia in the SDH of T1DM mice on D14 (Figures 5(f) and 5(g), *P* = 0.0003, *n* = 5). The Western blot assays showed that treatment with rIGF-1 attenuated the increase in the spinal protein expression of iNOS, IL-1*β*, and TNF-*α* but did not alter the Arg-1 expression (Figures 5(i) and 5(j), *P* = 0.0061 for iNOS, *P* = 0.4245 for Arg-1, *P* = 0.0007 for IL-1*β*, and *P* = 0.0015 for TNF-*α*, *n* = 5).

### 3.6. Treatment with EGCG Reduced Pain-Like Behaviors and Restored IGF-1/IGF1R Signaling in the Spinal Cord of Diabetic Mice

**Figure 6:**
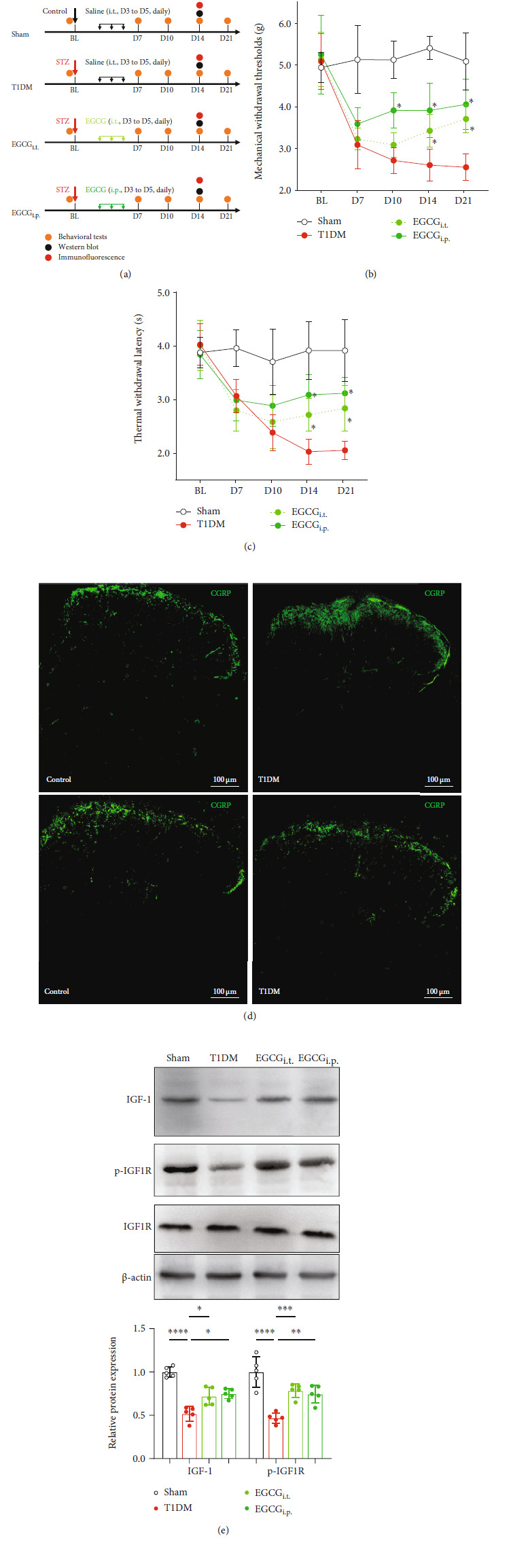
Treatment with EGCG reduced pain-like behaviors and restored IGF-1/IGF1R signaling in the spinal cord of diabetic mice. (a) Schematic illustration of the group assignment in the experimental stage. (b) i.t. (EGCG_i.t._, 2 *μ*g/d, i.t., from D3 to D5 after STZ injection) or i.p. (EGCG_i.p._, 20 mg/kg/d, i.p., from D3 to D5 after STZ injection) treatment with EGCG alleviated mechanical allodynia (increase in PMWTs) in diabetic mice. Data were presented as the mean ± SD. ^∗^*P* < 0.05 vs. the T1DM group. Two-way ANOVA followed by Bonferroni's post hoc test. *n* = 8/group. (c) i.t. (EGCG_i.t._) or i.p. (EGCG_i.p._) treatment with EGCG reduced thermal hypersensitivity (elevation in PTWL) in diabetic mice. Data were presented as the mean ± SD. ^∗^*P* < 0.05 vs. the T1DM group. Two-way ANOVA followed by Bonferroni's post hoc test. *n* = 8/group. (d) Fluorescent results showing CGRP (green) immune staining in the SDH of mice on D14. The nuclei were counterstained with DAPI (blue). Scale bar = 100 *μ*m. (e) Western blot showing the protein expression of IGF-1, p-IGF1R, and IGF1R in the lumbar spinal cord on D14. ^∗^*P* < 0.05,  ^∗∗^*P* < 0.01,  ^∗∗∗^*P* < 0.001, or^∗∗∗∗^*P* < 0.0001.*n* = 5/group. Data were presented as the mean ± SD. BL: baseline; CGRP: calcitonin gene-related peptide; EGCG: (−)-epigallocatechin-3-gallate; EGCG_i.p._: mice treated intraperitoneally with EGCG (20 mg/kg/d, from D3 to D5 after STZ injection); EGCG_i.t._: mice treated intrathecally with EGCG (2 *μ*g/d, from D3 to D5 after STZ injection); IGF1R: insulin-like growth factor 1 receptor; p-IGF1R: phospho-IGF1R (Tyr-1161); SDH: the spinal dorsal horn; T1DM: the mice treated with STZ (diabetic mice).

Although we have established the beneficial effect of strengthening IGF-1 signaling in diabetic mice, the application of rIGF-1 still lacks plausibility in the clinical setting. Thus, we intended to explore whether EGCG, the main component of green tea [48], could affect PDN by regulating IGF-1/IGF1R signaling. In this experimental stage, EGCG was injected i.t. (EGCG_i.t._, 2 *μ*g/d, i.t., from D3 to D5 after STZ injection) or i.p. (EGCG_i.p._, 20 mg/kg/d, i.p., from D3 to D5 after STZ injection) to the diabetic mice (Figure 6(a)). The behavioral results showed that both i.t. and i.p. treatment with EGCG induced an elevation in PMWT and PTWL (Figures 6(b) and 6(c); for PMWT, EGCG_i.t._ vs. T1DM, *F* (1, 14) = 22.42, *P* = 0.0003, EGCG_i.p._ vs. T1DM, *F* (1, 14) = 74.25, *P* < 0.0001, *n* = 8; for PTWL, EGCG_i.p._ vs. T1DM, *F* (1, 14) = 13.47, *P* = 0.0025, EGCG_i.p._ vs. T1DM, *F* (1, 14) = 27.17, *P* = 0.0001, *n* = 8). Furthermore, the intensified CGRP immunoreactivity in the SDH of T1DM mice was decreased by either i.t. or i.p. treatment with EGCG (Figure 6(d)). The Western blot results verified that the i.t. or i.p. treatment with EGCG improved the spinal protein levels of IGF-1 and p-IGF1R in diabetic mice (Figure 6(e), EGCG_i.t._ vs. T1DM, *P* = 0.0023 for IGF-1, *P* = 0.0011 for p-IGF1R, *n* = 5; EGCG_i.p._ vs. T1DM, *P* = 0.0007 for IGF-1, *P* = 0.0035 for p-IGF1R).

### 3.7. EGCG Attenuated Neuroinflammation and Prevented Microglia from Shifting towards Activated M1 Phenotype in the Spinal Cord of Diabetic Mice

**Figure 7:**
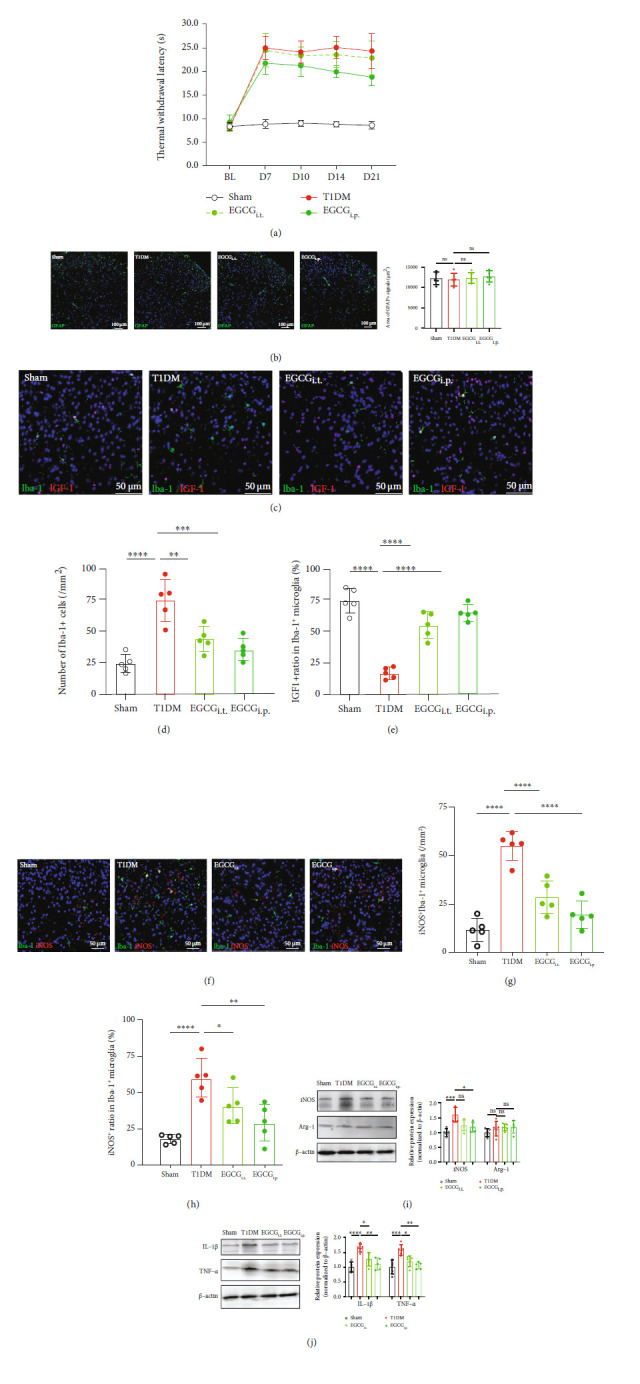
EGCG attenuated neuroinflammation and prevented microglia from shifting towards activated M1 phenotype in the spinal cord of diabetic mice. (a) Time course of blood glucose concentration in the sham, T1DM, EGCG_i.t._ (EGCG 2 *μ*g/d, i.t., from D3 to D5 after STZ injection), and EGCG_i.p._ (EGCG, 20 mg/kg/d, i.p., from D3 to D5 after STZ injection) groups. Data were presented as the mean ± SD. *n* = 8/group. (b) The area of GFAP immunoreactivity in the SDH of the sham, T1DM, EGCG_i.t._, and EGCG_i.p._ groups on D14. *n* = 5/group. Data were presented as the mean ± SD. (c) Fluorescent graphs displaying the double-labeling of IGF-1 (red) with Iba-1 (green) in the SDH of the sham, T1DM, EGCG_i.t._, and EGCG_i.p._ groups. Scale bar = 50 *μ*m. (d) The statistical analysis of Iba-1^+^ cells in the SDH of the sham, T1DM, EGCG_i.t._, and EGCG_i.p._ groups. *n* = 5/group. Data were presented as the mean ± SD. ^∗∗^*P* < 0.01 or ^∗∗∗^*P* < 0.001. (e) The analysis of IGF-1^+^ ratio in Iba-1^+^ microglia in the SDH of the sham, T1DM, and rIGF-1 groups. *n* = 5/group. Data were presented as the mean ± SD. ^∗∗∗∗^*P* < 0.0001. (f) Fluorescent graphs showing the double-labeling of iNOS (red) with Iba-1 (green) in the SDH of the sham, T1DM, and rIGF-1 groups. Scale bar = 50 *μ*m. (g) The statistical analysis of iNOS^+^Iba-1^+^ cells in the SDH of the sham, T1DM, EGCG_i.t._, and EGCG_i.p._ groups. *n* = 5/group. Data were presented as the mean ± SD. ^∗∗∗∗^*P* < 0.0001. (h) The analysis of iNOS^+^ ratio in Iba-1^+^ microglia in the SDH of the sham, T1DM, and rIGF-1 groups. *n* = 5/group. Data were presented as the mean ± SD. ^∗^*P* < 0.05,  ^∗∗^*P* < 0.01, or^∗∗∗∗^*P* < 0.0001. (i) Western blot showing the protein expression of iNOS and Arg-1 in the lumbar spinal cord on D14. ^∗^*P* < 0.05 or^∗∗∗^*P* < 0.001.*n* = 5/group. Data were presented as the mean ± SD. (j) Western blot demonstrating the protein expression of IL-1*β* and TNF-*α* in the lumbar spinal cord on D14. ^∗^*P* < 0.05,  ^∗∗^*P* < 0.01,  ^∗∗∗^*P* < 0.001, or^∗∗∗∗^*P* < 0.0001. *n* = 5/group. Data were presented as the mean ± SD. Arg-1: arginase 1 (M2 phenotype microglia marker); EGCG: (−)-epigallocatechin-3-gallate; EGCG_i.p._: mice treated intraperitoneally with EGCG (20 mg/kg/d, from D3 to D5 after STZ injection); EGCG_i.t._: mice treated intrathecally with EGCG (2 *μ*g/d, from D3 to D5 after STZ injection); IL-1*β*: interleukin 1*β*; iNOS: inducible nitric oxide synthase (M1 phenotype microglia marker); ns: not significant; TNF-*α*: tumor necrosis factor-*α*; SDH: the spinal dorsal horn; T1DM: the mice treated with STZ (diabetic mice).

We observed that the i.t. treatment with EGCG did not notably modify the blood glucose level of T1DM mice (Figure 7(a), *F*(1, 14) = 3.501, *P* = 0.0824, *n* = 8), whereas the mice injected i.p. with EGCG had a significantly lower blood glucose level than T1DM mice (Figure 7(a), *F*(1, 14) = 58.05, *P* < 0.0001, *n* = 8). Treatment with EGCG did not remarkably influence the GFAP immunoreactivity (Figure 5(b), *P* > 0.05, *n* = 5) but diminished Iba-1^+^ microglia in the SDH of T1DM mice on D14 (Figures 7(c) and 7(d), *P* < 0.01 or *P* < 0.001, *n* = 5). Moreover, i.t. or i.p. treatment with EGCG raised the IGF-1^+^ ratio in Iba-1^+^ cells (Figures 7(c) and 7(e), *P* < 0.0001, *n* = 5) and decreased iNOS^+^Iba-1^+^ microglia in the SDH of T1DM mice on D14 (Figures 5(f) and 5(g), *P* < 0.0001, *n* = 5). The Western blot assays showed that i.t. or i.p. treatment with EGCG blocked the elevation in the spinal protein expression of iNOS, IL-1*β*, and TNF-*α* but did not alter the Arg-1 expression (Figures 5(i) and 5(j), *P* < 0.05 or *P* < 0.01, *n* = 5).

## 4. Discussion

Our study identified the dysfunction of IGF-1 signaling, especially microglial IGF-1, as an essential mechanism underlying the spinal neuroinflammation in PDN. We also revealed the potency of ECGG in maintaining microglia IGF-1 signaling and reducing neuroinflammation in PDN. Specifically, we found that the spinal IGF-1 expression markedly declined parallel to the development of pain-like behaviors, neuroinflammation (increased IL-1*β*, TNF-*α*, and Iba-1^+^ microglia), and the increased M1 microglia polarization (increased iNOS^+^Iba-1^+^ microglia) in diabetic mice. In the spinal cord, IGF-1 could colocalize with neurons, astrocytes, and microglia, but only microglial IGF-1 was suppressed in T1DM mice. Furthermore, we found that i.t. administration of rIGF-1 as well as i.t. or i.p. treatment with EGCG alleviated the diabetes-induced pain-like behaviors, reduced neuroinflammation, averted the M1 microglia polarization, and recovered the microglial IGF-1 expression.

Neuroinflammation served a significant role in peripheral nerve injury-induced neuropathic pain [49, 50]. Increasing evidence reveals that nonneuronal glia cells contribute largely to the neuroinflammation in the key region regulating pain (i.e., the SDH) [49, 50]. Our recent study found a significant rise in reactive astrocytes and microglia in the animal model of chronic constriction injury of the sciatic nerve [35]. However, studies have demonstrated a particular role of microglia activation in the SDH of diabetic animals, signifying a characteristic role of microglia in PDN. This was verified in the present study that the GFAP^+^ immunoreactivity was not altered while Iba-1^+^ microglia were extensively increased in the SDH of diabetic mice.

IGF-1 is a protein that has various functions in the CNS, including neuronal survival and synapse growth [16]. Generally, IGF-1 is considered a neuroprotective molecule which is at least partly ascribed to its anti-inflammatory property [22]. Consistent with this, a decrease in IGF-1 signaling has been related to neurodegeneration, depressive disorders, and other brain diseases [22]. Also, intrathecal administration of IGF-1 could induce a central antinociceptive effect and reduce neuroinflammation in the spinal cord of normal rats [51]. This had led us to speculate a potential role of IGF-1 for treating PDN and the associated spinal neuroinflammation.

As expected, we observed that IGF-1 signaling diminished in the spinal cord of T1DM mice, as indicated by the decrease in the protein expression of IGF-1 and p-IGF1R. The downregulation of spinal IGF-1 signaling might be associated with the inflammatory reaction, as the compromised spinal IGF-1 signaling was accompanied by the induction of neuroinflammation (increased IL-1*β*, TNF-*α*, and Iba-1^+^ microglia) and the intensified M1 microglia polarization (increased iNOS^+^Iba-1^+^ microglia). We further corroborated the relationship by displaying that the intrathecal administration of rIGF-1 alleviated pain-like behaviors and neuroinflammation in the spinal cord of diabetic mice. These results robustly suggested a potential beneficial effect of maintaining spinal IGF-1 signaling by suppressing neuroinflammation.

Previous studies have reported that IGF-1 can express in neurons, astrocytes, and microglia in the human brain, and its main source is microglia [22]. Indeed, we found that IGF-1 immunostaining colocalized with neurons, astrocytes, and microglia, but the largest proportion was astrocytes rather than microglia in the lumbar spinal cord of diabetic mice. These results are consistent with our recent animal studies in nerveinjury-induced neuropathic pain [35] and chemotherapy-induced peripheral neuropathy [36]. However, we noted that merely microglial IGF-1 was significantly suppressed in diabetic mice while the alterations in neurons and astrocytes were absent, implying a critical role of impaired microglial IGF-1 signaling underlying the pathogenesis of PDN.

IGF-1 has long been recognized in regulating microglial phenotypes [22, 24]. Some studies indicate that IGF-1^+^ microglia represent the anti-inflammatory M2 phenotype [22, 24], while others argue that IGF-1 could be present in resting (M0) microglia [52]. In this regard, we detected microglial M1 and M2 phenotypes by immunofluorescence in the spinal cord and showed that the main proportion of activated Iba-1^+^ microglia were the M1 phenotype (iNOS^+^) while the M2 microglia (Arg-1^+^) were rare in the SDH of either control or diabetic mice. Additional Western blot data verified the result by demonstrating that spinal iNOS protein expression was markedly increased in diabetic mice but without obvious alteration in Arg-1. Combined with the observed decrease of IGF-1^+^ microglia in diabetic mice, we thought that IGF-1^+^ microglia were more likely to denote the resting microglia rather than the M2 phenotype. This result is in agreement with a study on spinal cord injury and experimental autoimmune encephalomyelitis, which demonstrated that spinal Arg-1^+^ cells are not resident but mainly derived from macrophages migrating from responsive immune organs [53].

Although we had revealed the benefit of rIGF-1 supplementation for PDN, caution is still needed for expanding its applications in neuropathic pain. One of our current studies showed that IGF-1 signaling overactivation contributed to the development of neuropathic pain caused by peripheral nerve injury [35], implying that overdose rIGF-1 might even aggravate PDN. EGCG, a potent component of green tea, has been reported with robust ant-inflammatory effects in the CNS [27, 28]. Some lines of evidence also unveiled an antidiabetes property and antiobesity effect of EGCG [29–31], making it a suitable candidate for treating spinal neuroinflammation in PDN. Our results showed that i.t. treatment with EGCG obtained a comparable effect as rIGF-1 in reducing neuroinflammation (decreased IL-1*β*, TNF-*α*, and Iba-1^+^ microglia). Besides, i.t. administration of EGCG attenuated the inhibition of spinal IGF-1 signaling and enhanced microglia IGF-1 expression in diabetic mice. These results contrast the previous study in cancer disease that EGCG inhibited the proliferation of cancer cells by suppressing IGF-1 signaling [32]. This phenomenon may be explained by different pathological conditions: IGF-1 is overexpressed in cancer cells as a promitogenic molecule, while under the diabetic condition, spinal IGF-1 signaling is much below the normal range. Moreover, i.t. EGCG treatment prevented microglia polarization (reduced iNOS^+^ microglia) and maintained IGF-1^+^ microglia (putative resting microglia) in diabetic mice, highlighting the potency of EGCG in balancing microglial polarization. This is consistent with previous findings that delayed EGCG treatment repressed microglial M1 polarization and attenuated neuroinflammation in the ischemic brain [27].

Although i.t. supplementation of rIGF-1 is beneficial for diabetic mice, the invasiveness of intrathecal injection might limit its application in the clinical setting. Hence, we also assess whether systemic administration (i.p.) of EGCG can provide a comparable beneficial outcome. Our data showed that i.p. injection of EGCG accomplished similar antinociceptive and anti-inflammatory effects as the i.t. injection. We thought that this might be attributed to the dual effects of EGCG on peripheral (DRG) or central neurons. The pathology of PDN includes the peripheral and central mechanism: diabetes/hyperglycemia causes substantial peripheral nerve injury and DRG neuronal dysfunction, which subsequently result in the alteration in the spinal cord. As EGCG is able to cross the blood-brain barrier [54], the i.p.-treated EGCG might partially infiltrate into the spinal cord while the other remained in the peripheral to affect the DRG function, collectively conducting the antinociceptive effect. Thus, we believed that systemic treatment with EGCG could be a promising approach for treating clinical PDN.

In conclusion, our data highlighted the significance of preserving spinal IGF-1 signaling in treating microglia-related neuroinflammation in PDN. This study also provides novel insights into the neuroprotective mechanisms of EGCG against neuropathic pain and neuroinflammation through IGF-1 signaling, indicating that this agent or habitual tea intake would be a favorable treatment for diabetic patients.

## Figures and Tables

**Figure 8:**
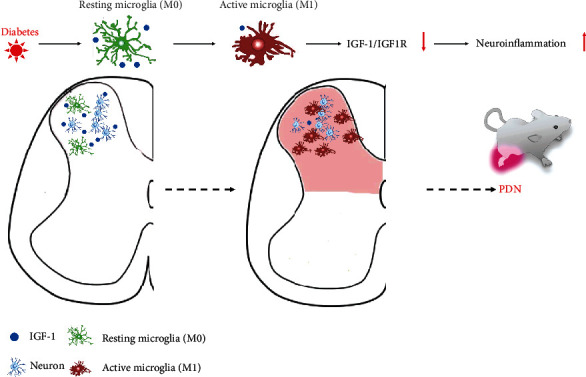
Schematic illustrating the mechanism of IGF-1 signaling and EGCG in combating spinal neuroinflammation and the pathogenesis of PDN. EGCG: (−)-epigallocatechin-3-gallate; IGF-1: insulin-like growth factor 1; IGF1R: insulin-like growth factor 1 receptor; PDN: painful diabetic neuropathy.

## Data Availability

The datasets during and/or analyzed during the current study are available from the corresponding author on reasonable request.

## Abbreviations

Arg-1: Arginase 1 (M2 phenotype microglia marker)
BL: Baseline
DAPI: 4′,6-Diamidino-2-phenylindole
EGCG: (−)-Epigallocatechin-3-gallate
GFAP: Glial fibrillary acidic protein
Iba-1: Ionized calcium-binding adaptor protein-1
IGF-1: Insulin-like growth factor 1
IGF1R: Insulin-like growth factor 1 receptor
IL-1*β*: Interleukin 1*β*
iNOS: Inducible nitric oxide synthase (M1 phenotype microglia marker)
PDN: Painful diabetic neuropathy
PMWT: Paw mechanical withdrawal threshold
PTWL: Paw thermal withdrawal latency
SDH: The spinal dorsal horn
STZ: Streptozotocin
T1DM: Type 1 diabetes mellitus
TNF-*α*: Tumor necrosis factor-*α*.
